# Cancer treatment in childhood and testicular function: the importance of the somatic environment

**DOI:** 10.1530/EC-17-0382

**Published:** 2018-01-19

**Authors:** Jan-Bernd Stukenborg, Kirsi Jahnukainen, Marsida Hutka, Rod T Mitchell

**Affiliations:** 1 NORDFERTIL Research Lab StockholmPediatric Endocrinology Unit, Department of Women’s and Children’s Health, Karolinska Institutet and University Hospital, Stockholm, Sweden; 2 Division of Haematology-Oncology and Stem Cell TransplantationChildren’s Hospital, University of Helsinki, Helsinki University Central Hospital, Helsinki, Finland; 3 MRC Centre for Reproductive HealthThe Queen’s Medical Research Institute, The University of Edinburgh, Edinburgh, UK; 4 Edinburgh Royal Hospital for Sick ChildrenEdinburgh, UK

**Keywords:** fertility, testis, cancer treatment, fertility preservation

## Abstract

Testicular function and future fertility may be affected by cancer treatment during childhood. Whilst survival of the germ (stem) cells is critical for ensuring the potential for fertility in these patients, the somatic cell populations also play a crucial role in providing a suitable environment to support germ cell maintenance and subsequent development. Regulation of the spermatogonial germ-stem cell niche involves many signalling pathways with hormonal influence from the hypothalamo-pituitary-gonadal axis. In this review, we describe the somatic cell populations that comprise the testicular germ-stem cell niche in humans and how they may be affected by cancer treatment during childhood. We also discuss the experimental models that may be utilized to manipulate the somatic environment and report the results of studies that investigate the potential role of somatic cells in the protection of the germ cells in the testis from cancer treatment.

## Introduction

Fertility in males is dependent on the presence of a germ cell population that is capable of developing into sperm in adulthood. However, the ability of spermatogonial stem cells (SSC) to give rise to sperm is dependent on the presence of functioning somatic cell populations. This unique testicular microenvironment which includes the SSC, supporting somatic cell populations and the presence of specific growth factors is known as the germ-stem cell niche. Cell populations deemed to be important include Sertoli, peritubular myoid and Leydig cells, with contribution from additional interstitial cell types and the vasculature (1). Animal studies, largely conducted in rodents, have identified a number of signalling pathways involved in maintenance of the germ-stem cell niche and highlighted their impacts on fertility when these pathways are disrupted (reviewed in (2, 3)).

In humans, cancer and its treatment are a recognised cause of subsequent infertility. This may be as a result of the underlying cancer (e.g. testicular cancer) or due to the damaging effects of chemotherapy or radiotherapy (4). Treatments that directly damage the SSC population will impact on subsequent fertility; however, indirect effects on germ cells mediated through the somatic cell populations may also lead to infertility (5). For adult men with cancer, it is possible to store sperm prior to treatment to allow them to be able to father children in the future using artificial reproductive technologies. However, for those who are likely to be rendered infertile by their treatment and are unable to produce mature sperm (e.g. prepubertal boys), there are currently no established options to allow them to father biological children of their own (6). Several approaches are being investigated to preserve or restore fertility in these patients and whilst the primary focus of the majority of these studies are on the germ cells, the role of the somatic cell populations in mediating the effects of cancer treatment on the testis or in supporting the restoration of fertility following treatment is poorly understood (5).

In this review, we will describe the development and function of the key somatic cell populations in the testis and how they may be affected by cancer and its treatment. We will describe the experimental models that can be used to assess somatic cell function and discuss studies that have attempted to preserve fertility in males, which may involve manipulation of the somatic cells in the testis. We will primarily focus on studies involving human and non-human primates, supported by findings from rodent studies where appropriate.

## Testicular development in infancy, childhood, puberty and adulthood

### Hormonal control of testicular development and function

**Figure 1:**
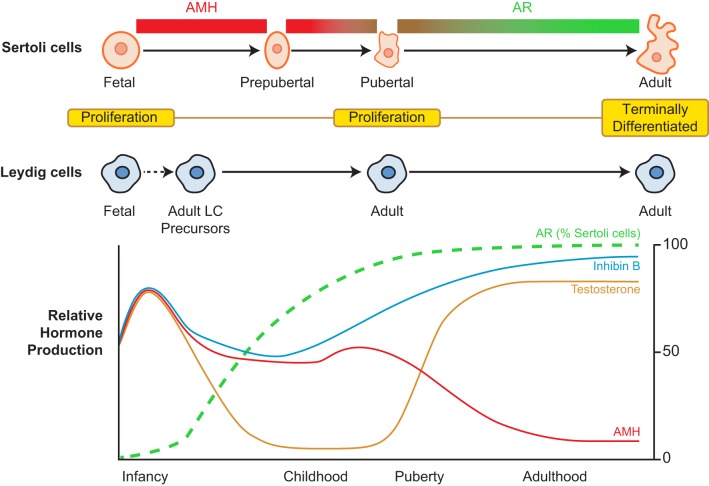
Sertoli and Leydig cell development and profile of reproductive hormone secretion in humans from birth to adulthood. Sertoli cell maturation involves changes in morphology, protein expression (including AMH and AR) and proliferation, whilst Leydig cell development involves two distinct populations of cells, which include a foetal Leydig cell population which regresses postnatally to be replaced by an adult Leydig cell population derived from a precursor population present in the prepubertal testis. Relative hormone production based on data taken from normal human populations (19, 20, 21). Whilst gonadotrophins are undetectable during childhood, Sertoli cell-derived hormones Inhibin B and AMH remain detectable.

Secretion of gonadotrophins, luteinising hormone (LH) and follicle-stimulating hormone (FSH), from the pituitary gland is responsible for regulating hormonal control of the testis in the male. LH and FSH signal through the testicular somatic Leydig and Sertoli cell populations respectively. The male hypothalamo-pituitary-gonadal (HPG) axis is active in humans from foetal life and during the early postnatal period. This pattern of secretion has also been demonstrated in many other non-human primates, including the rhesus monkey and marmoset (7, 8). In humans and non-human primates after the rise in gonadotrophins and testosterone during early infancy, there follows a period of relative HPG quiescence during which levels of these hormones are suppressed (Fig. 1). This ‘childhood period’ lasts from the end of infancy until peri-puberty (8). Although childhood has been described as a quiescent period in terms of testicular activity, it is clear that there is activity occurring within the testis, which includes periods of germ cell proliferation (9) and the transient appearance of meiotic cells (10). Puberty heralds the reactivation of the HPG axis, and this activity remains throughout adulthood. Both LH and FSH indirectly influence germ cell development in the testis. LH binds to the LH/CG receptor to promote testosterone secretion from the Leydig cells, and FSH signals through the FSH receptor of Sertoli cells within the seminiferous tubules to support spermatogenesis. This emphasises the importance of the somatic cells in supporting spermatogenesis and highlights the potential for targeting somatic cells for the purposes of preserving fertility in cancer patients.

### Germ cells – development, maturation and function

Future fertility requires normal development of the germ cell population from foetal life through to adulthood. Foetal gonocytes must undergo differentiation to prespermatogonia during late foetal and early postnatal life (11) and spermatogonial proliferation continues during childhood. In humans, SSC have been reported to be present in the testis from ~2 to 3 months of postnatal age (11, 12). From puberty, SSCs exhibit a fine balance between self-renewal, which maintains their numbers and differentiation to generate meiotic cells to produce spermatozoa (13). Two populations of SSCs have been described in primates, A_dark_ SSCs have been proposed to represent a regenerative reserve that may be replenished following insult e.g. cancer treatment (14), whilst A_pale_ SSCs are also present in the postnatal testis and are proposed to represent a progenitor population acting as a functional reserve (14). In the rhesus monkey, A_pale_ spermatogonia form clones and the subsequent differentiation and proliferation occurs in synchrony within these clones (15). Whilst differences in proliferation of the A_dark_ (slow cycling) and A_pale_ populations have been described in steady-state conditions, this may not be the case following damage by cancer treatment. Understanding of spermatogonial development in humans remains limited; however, it is clear that there are key differences between the spermatogonial populations of the primate and rodent testis, which make direct extrapolation from the results of rodent studies challenging (reviewed in (14)).

### Sertoli cells – development, maturation and function

Sertoli cells serve a number of important functions in the testis including support of spermatogenesis by mediating FSH signalling. This, combined with their location within the seminiferous tubules and close association to the SSC means that Sertoli cells are widely considered to be a key component of the SSC niche. The total number of Sertoli cells determines the number of germ cells that can be supported and hence maximum sperm output (16). Sertoli cell proliferation in humans occurs during foetal and early postnatal life. Sertoli cells cease proliferation during childhood until a second proliferative wave begins around puberty (16). Interestingly, the marmoset monkey does not exhibit a prepubertal period of Sertoli cell proliferation; however, this does occur in marmosets in which Sertoli cell proliferation is suppressed during the neonatal period with GnRH antagonists. This indicates a compensatory increase in Sertoli cell number to normal adult levels, which may be important in terms of recovery of the Sertoli cell population following cancer treatment (17). Once adulthood has been reached, Sertoli cells have ceased to proliferate and no further compensation of Sertoli cell number is possible (18). Sertoli cell function during childhood may be indicated by the presence of normal levels of anti-Mullerian hormone (AMH) and inhibin B during a period when measurement of gonadotrophins may not be helpful to assess the testicular function due to the quiescence of the HPG axis (19, 20, 21); however, the role of inhibin B and AMH in determining the function of the germ-stem cell niche following damage has not been elucidated in prepuberty (Fig. 1).

Sertoli cell maturation is important for the support of spermatogenesis, and immaturity of Sertoli cells is a hallmark of many conditions that impair male fertility in humans (22, 23). Importantly, androgen action through Sertoli cells has been shown to be required for fertility in mice, and responsiveness to androgens requires the presence of the androgen receptor (AR). Knockout of AR specifically in Sertoli cells of mice results in azoospermia and infertility (24). Androgen receptor is not expressed in the Sertoli cells of the foetal or early postnatal human testis; however, AR can be identified in an increasing proportion of Sertoli cells during childhood before the final maturation of Sertoli cells (16, 25, 26) (Fig. 1). Terminal differentiation into a mature Sertoli cell involves changes in cellular morphology and protein expression, including downregulation of AMH, resulting in a non-mitotic mature Sertoli cell capable of supporting spermatogenesis (27). Sertoli cell maturation is also associated with the development of tight junctions that create the blood-testis barrier, which divide the seminiferous tubule into two distinct compartments. The spermatogonia (including the SSC population) remain in the basal compartment in adulthood, separated from the differentiating germ cells in the adluminal compartment. Whilst Sertoli cells appear to be important for supporting germ cell differentiation and spermatogenesis in adult mice, they are also important for regulating factors (e.g. retinoic acid) that prevent premature meiotic development in germ cells of the foetal testis, thus supporting the development of pre-spermatogonia (28, 29). In mice, this includes effects on the retinoic acid pathway (e.g. CYP26B1, NANOS2) (28, 29, 30); whilst in humans, additional factors (e.g. DMRT1) have also been proposed to play a role in regulating meiotic entry (31).

SSC and Sertoli cells remain in close contact with the basement membrane, which contains a number of extracellular matrix proteins potentially involved in promoting SSC development. Factors such as β1 integrin (expressed by spermatogonia), which associate with laminins in the basement membrane have been shown to be important for spermatogenesis in rodents (32), indicating that the basement membrane is also likely to be a key component of the SSC niche.

### Peritubular myoid cells – development, maturation and function

The role of the peritubular myoid cell in supporting spermatogenesis in human and non-human primates is not well characterised and much of the understanding is derived from rodent studies. In mice, androgen action specifically through peritubular myoid cells has been shown to be necessary for fertility. Mice in which AR was knocked-out specifically in peritubular myoid cells were azoospermic and infertile (33). Androgen signalling through peritubular myoid cells also occurs in humans with AR expression described in peritubular myoid cells from foetal life (34, 35). Furthermore, inhibition of tyrosine kinase signalling in immature rats has been shown to impair peritubular myoid cell proliferation with subsequent reduction in adult testis size (36); however, the importance of these signalling pathways in PTM cells for subsequent testicular development and fertility in humans is unknown.

### Leydig cells – development, sub-populations, maturation and function

**Figure 2:**
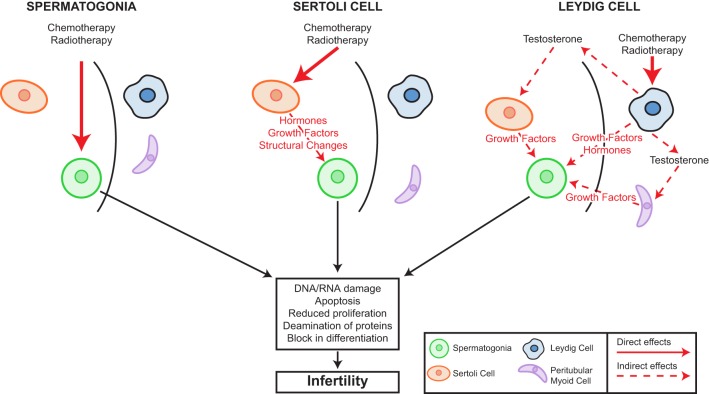
Cellular targets for chemotherapy and/or radiotherapy-induced damage in the prepubertal testis. Infertility may result from damage within the seminiferous tubules as a result of direct damage to the spermatogonia leading to alterations in proliferation, differentiation, protein deamination and apoptosis and ultimately infertility. Alternatively, damage to the Sertoli cells by such treatments may result in alterations in hormones, growth factors or seminiferous tubule structure that will indirectly mediate the effects of chemo/radiotherapy on the germ cells. Similarly, interstitial effects include damage to the Leydig cells that can lead to alterations in hormones or growth factors that may impact germ cells directly or indirectly (e.g. testosterone deficiency) through effects other somatic cell populations.

Leydig cells are located within the interstitium of the testis and are responsible for the production of key hormones including testosterone and insulin-like growth factor 3 (Insl3). Insl3 is involved in the trans-abdominal phase of testicular descent, which is important for subsequent testicular function, whilst testosterone is important for the final stage of testicular descent, masculinisation and spermatogenesis (37). Distinct populations of Leydig cells have been described at different stages during life. In the human, the foetal Leydig cell population regresses towards the end of the first year of life, whilst a population of undifferentiated mesenchymal cells remain in the interstitium. At puberty, these cells begin to proliferate and differentiate to adult testosterone-producing Leydig cells. Once terminally differentiated, adult Leydig cells cease proliferation (38). Leydig cells have been proposed as key components of the SSC niche based on several observations from studies in mice. This includes the location of SSC on the basement membrane in close proximity to the interstitium, in addition to Leydig cell-produced testosterone supporting spermatogenesis through AR signalling in Sertoli and peritubular myoid cells (38) (Fig. 2).

In addition to Leydig cells, the interstitium contains the vasculature, which has also been described as an important component of the SSC niche. The spatial location of the blood vessels in close proximity to the SSC in mice supports this hypothesis (1).

### Somatic cell signalling pathways for supporting spermatogenesis

Several signalling pathways have been shown to be involved in somatic cell support for SSC development in rodents. This includes GDNF, which is expressed in the Sertoli cells and peritubular cells of the mouse testis and signals through the GFRα1 receptor on SSC. GDNF signalling has been proposed to be important for SSC self-renewal based on a study that demonstrated that manipulation of GDNF signalling appears to alter the balance between SSC self-renewal and differentiation, thereby resulting in the depletion of the SSC pool or alternatively the failure of SSC differentiation towards meiosis (39). However, whilst GDNF has a general role in maintaining SSC proliferation and survival, several other somatic cell-derived factors have been shown to be important in self-renewal of SSC in *in vitro* systems, including colony-stimulating factor 1 (CSF-1; Leydig cells, peritubular myoid cells), fibroblast growth factor 2 (FGF2; Sertoli cells), epidermal growth factor (EGF; Sertoli cells), insulin-like growth factor 1 (IGF1; Sertoli cells, Leydig cells) and leukaemia inhibitory factor (LIF; Sertoli, Leydig cells) (2, 40, 41, 42). Migration of the germ cells from the centre to the basement membrane of the seminiferous tubules is important for subsequent spermatogenesis, and this process has been shown to be attenuated by loss of Sertoli cell factors such as GATA4 (3). GATA4 appears to play a role in maintenance of the SSC niche through regulation of chemokine signalling such as Sertoli cell-derived CXCL12 (3), which has also been shown to be impaired in other models in which there is failure of prospermatogonial migration, such as the Sin3a-knockout mouse (43).

Whilst rodent studies have uncovered a number of SSC niche signalling pathways that can affect SSC self-renewal and differentiation, whether the same mechanisms are also important for SSC development in humans and whether manipulation of these pathways can prevent SSC loss or enhance SSC survival and differentiation in the context of exposure to cytotoxic therapies is unknown.

## Effects of gonadotoxic therapies on the prepubertal testis – evidence from human and non-human primate studies

Testicular cells including the germ and somatic populations are sensitive to cytotoxic treatment such as chemotherapy and radiotherapy. Whilst fertility is ultimately dependant on the development of mature gametes from undifferentiated germ cells, infertility may result directly from damage to the germ cells or indirectly via damage to the somatic population. Moreover, somatic cell damage may affect germ cells by a number of mechanisms including paracrine (such as those described earlier) and endocrine signalling pathways (Fig. 2).

### Germ cell effects – direct

Low doses of chemotherapy or radiotherapy may deplete the pool of differentiating spermatogonia, whilst reserve SSCs survive, and spermatocytes and spermatids continue their maturation into sperm (44). The potential for recovery of sperm production after a cytotoxic insult in adulthood or at puberty depends on the ability of mitotically quiescent stem spermatogonia to survive and resume mitotic activity and to produce differentiating spermatogonia. If the damage is severe, for example, as a result of a high cumulative dose of alkylating agent or irradiation (45), all the A_dark_ SSCs may commit to apoptosis and the patient will become permanently infertile. Spermatogonia have been shown to be susceptible to such depletion at all stages of life (46, 47).

Alkylating and platinum agents cause direct DNA and RNA damage and can therefore affect even non-dividing reserve (A_dark_) stem cells. The threshold dose of cyclophosphamide, in relation to infertility, has been shown to be between 7.5 and 10 g/m^2^ (48, 49, 50). However, a recent large study of non-irradiated childhood cancer survivors failed to identify any threshold dose for alkylating agent exposure that predicted impaired spermatogenesis or azoospermia after a median follow-up of 21 years (51). There may be other factors, in addition to absolute doses and regimen, such as genetic variation in drug metabolising pathways that modulate the impact of alkylating agent exposure on spermatogenesis or its recovery (51).

The germinal epithelium is very susceptible to irradiation-induced damage (52, 53). The progenitor and differentiating spermatogonia are radiosensitive to scattered doses as low as 0.1 Gy leading to short-term cessation of spermatogenesis (54). Doses of 2–3 Gy also affect stem cell spermatogonia and cause long-term azoospermia. Doses in excess of 6 Gy are able to deplete the SSC pool and lead to permanent infertility (54, 55). Fractionation of radiotherapy increases the germ cell toxicity possibly because of repeated hits to activated A_dark_ SSCs (55, 56). Total body irradiation (TBI), as conditioning for haematological stem cell transplantation (HSCT), is also associated with significant germ cell failure (57, 58). Following treatment with TBI (10 or 13 Gy), azoospermia was found in 85% of men and oligozoospermia occurred in the rest (59). Recovery of spermatogenesis never occurred before the 4th year after transplantation; therefore, azooospermia after HSCT may be overestimated if semen samples are evaluated too early.

### Sertoli cell effects

The underlying mechanisms of chemotherapy and irradiation-dependent germ cell loss are poorly understood in humans, and in particular, the effect on the testicular stem cell niche and the involvement of Sertoli cells. Evidence for Sertoli cell dysfunction following conditioning treatment for HSCT has been demonstrated by a typical pattern of raised FSH, low inhibin B +/− low AMH at puberty (60) and raised FSH has been shown to be a predictor of azoospermia in childhood cancer survivors (61, 62). Few clinical and experimental studies have been performed previously to explore direct effects of cancer treatments on the somatic compartment of the SSC niche and long-term recovery of spermatogenesis (47, 58, 63). The study of de Rooij using rhesus monkeys showed that single or fractionated irradiation with doses of 4–8.5 Gy leads to a dose-dependent increase in the proportion of seminiferous tubules, which are fully depleted of germ cells (63). A complete Sertoli cell only (SCO) situation was only observed at the highest single dose or following fractionated doses, whilst lower doses induced a mild-to-severe focal SCO pattern. This study reported a depletion of Sertoli cells at higher doses of irradiation leading to lower testis weights in adulthood. The study of Jahnukainen and coworkers showed that testicular irradiation with a single fraction of 10 Gy before initiation of pubertal testis growth in rhesus monkeys had a more severe detrimental effect on pubertal outgrowth of seminiferous tubules compared to irradiation of testes, which had started pubertal development (47). Interestingly, Sertoli cells before initiation of pubertal spermatogenesis were more radiosensitive than the Sertoli cells after initiation of spermatogenesis. These observations suggest that signals responsible for the terminal differentiation of the primate Sertoli cells at puberty affect the radiosensitivity of Sertoli cells. The authors speculated that since the increase in the amount of germ cells in the germinal epithelium at puberty is known to follow a peri-pubertal period of Sertoli cell proliferation, (64), the higher fraction of proliferating Sertoli cells in the peri-pubertal testis could have increased the proportion that are sensitive to irradiation. Pubertal status in humans at the time of HSCT has been shown to be an independent predictor of adult testicular volume (58), which in turn is primarily determined by Sertoli cell number (16). Exposure to gonadotoxic conditioning (typically TBI 10–12 Gy) before initiation of pubertal maturation led to significantly smaller adult testicular volumes (mean 9 mL) compared with HSCT during or after puberty (mean 14 mL) (58). This observation is consistent with the experimental data from monkeys and confirms that testicular irradiation before spermarche is more detrimental on outgrowth of seminiferous tubules and adult testicular volume than irradiation at puberty. Chemotherapy has also been reported to affect the Sertoli cell population as determined by the presence of undifferentiated Sertoli cells in regions of impaired spermatogenesis in the adult testis, following chemotherapy exposure during childhood (65). Taken together, these findings provide evidence for effects of cancer treatment on the Sertoli cell population.

### Leydig cell effects

Chemotherapy-induced Leydig cell failure resulting in androgen insufficiency and requiring testosterone replacement therapy is relatively rare (66). The majority of males treated for cancer undergo a normal puberty and most produce normal adult levels of testosterone. Compensated Leydig cell failure (increased LH with low normal testosterone levels or exaggerated FSH and LH responses to LH-releasing hormone) and gynaecomastia have been reported in patients treated with a combination of mustine and procarbazine and after treatment with high-dose cyclophosphamide (49, 67, 68). Young boys and adolescent males who receive 200 mg/kg (6.7 g/m^2^) cyclophosphamide or a combination of busulphan and cyclophosphamide as conditioning therapy for bone marrow transplantation appear to retain normal Leydig cell function (57). Whilst relatively low irradiation doses may result in damage to the seminiferous epithelium, resulting in oligozoospermia (69), much higher doses (>20 Gy) appear to be required to cause Leydig cell dysfunction. However, significant rises in LH have been demonstrated following single radiation doses above 0.75 Gy and fractionated doses above 2 Gy (70). No change in testosterone levels was seen at these doses, indicating compensated Leydig cell damage, and LH values gradually return to normal levels over 30 months. Essentially all males who are pubertal or younger when they receive 24 Gy for testicular leukaemia are at high risk of delayed sexual maturation associated with decreased testosterone levels and require androgen replacement therapy (52, 53). The majority of males who receive 20 Gy fractionated testicular irradiation appear to retain their ability to produce normal amounts of testosterone (57, 66). Whilst testosterone production is generally maintained in patients receiving cancer treatment, whether there are additional aspects of Leydig cell function that may impact on germ cells in the prepubertal testis, and whether they can be affected by cancer treatment is unknown.

### Peritubular myoid cell effects

Peritubular myoid cells are increasingly recognised as important players in the regulatory network of testicular somatic cells and the stem cell niche (35). One recent study has explored irradiation-induced changes in peritubular myoid cells in testicular tissue from immature non-human primates (71). In this study, testis tissue was xenografted into immunocompromised host mice. Smooth muscle actin (SMA; a functional marker of PTM cells) expression in non-irradiated tissues was reported to appear following the 6.5 months of xenografting indicating cell differentiation. However, in irradiated grafts, the appearance of SMA was partly or almost fully diminished. Similarly, normal expression of signalling of chemokine ligand type 11 (CXCL11) was only established in peritubular myoid cells of non-irradiated grafts. These findings suggest that irradiation can affect peritubular myoid cell development. However, it remains to be elucidated whether irradiation has a direct effect on peritubular myoid cells or if irradiation-evoked changes in SMA and CXCL11 is a consequence of indirect effects on the microenvironment. Furthermore, whether these peritubular myoid cell effects can impact on germ cell development remains to be determined.

### Vasculature effects

Radiation-induced damage to the vasculature has been recognised for several decades and exposure to radiation can lead to early intimal signs of atherosclerosis (72). Small vessels have been reported to be sensitive to high-dose radiotherapy and show subendothelial connective tissue proliferation, disruption of the elastic lamina, accumulation of intimal and subintimal fibrinoid substances, degeneration of smooth muscle, dense fibrosis of the adventitia, aggregates of foamy histiocytes in the damaged wall and eventual obliteration of the vasa vasorum. These changes are pathologically indistinguishable from naturally occurring atherosclerosis (73, 74). One clinical study evaluated the testicular blood flow by Doppler ultrasound in 12 subjects with non-obstructive azoospermia (including four patients who had received radiotherapy) and in patients with obstructive azoospermia (75). Testicular ultrasound in non-obstructive azoospermia revealed decreased or absent intratesticular arterial flow, whilst in obstructive azoospermia the testes exhibited a uniform perfusion comparable to controls. Whilst these effects on the vasculature are described in adults undergoing cancer therapy, no similar studies have been conducted in the prepubertal testis.

## Experimental approaches to fertility preservation – focus on somatic cell function

One approach to preserve fertility in young males who are due to receive treatment for cancer is to remove testicular tissue prior to treatment and to develop strategies to restore fertility using this tissue. Experimental models designed for generating gametes from immature testicular tissue in human and non-human primate primarily focus on the differentiation status of the germ cells. These studies can be broadly divided into three approaches; (a) germ (stem) cell transplantation; (b) tissue fragment transplantation and (c) *in vitro* culture employing single cells or tissue fragments. Although the somatic environment present in the testis plays an important role in the outcome of the spermatogenic process, the suitability of these systems for sustaining somatic cell development is often only mentioned briefly as additional information to the germ cell differentiation potential of the described system. Here, we will describe these experimental approaches focusing on development and function of somatic cell populations.

### Germ-stem cell transplantation

Germ cell transplantation has been successfully used in rodent models to generate gametes from germ cells derived from SSCs injected into the seminiferous tubules of germ cell-depleted host mice (76). The somatic cells are likely to play a key role in supporting the development of transplanted cells. This has been demonstrated in studies investigating the role of Sertoli cell-derived stem cell factor which signals via the cell surface *KIT* receptor on germ cells. Mutation in the gene encoding stem cell factor results in a failure of spermatogonial differentiation, which is rescued when the spermatogonia from these mice are transplanted into host mice without the mutation (77). These results highlight the importance of the somatic cell environment of the host mouse for supporting the development of SSC following transplant. This concept is supported by studies involving reciprocal experiments of transplantation of rat SSC into a host testis in which either the transplanted germ cells or the host testis has been irradiated. Transplantation of irradiated prepubertal SSC into a non-irradiated host mouse resulted in restoration of spermatogenesis, whereas transplantation of non-irradiated SSC into an irradiated adult rat testis did not result in resumption of spermatogenesis in the host (78).

Despite the success of transplanting SSC from rodents into host mice, transplant of germ cells from several other species including non-human primate failed to result in full spermatogenesis despite colonisation of mouse seminiferous tubules by the injected cells (79). Human SSCs transplanted into mice were also able to colonise the seminiferous tubules of a mouse host for at least 6 months (80). Proliferation of the spermatogonia occurred; however, no evidence of meiotic progression was demonstrated in this study or in another study using host mice lacking endogenous germ cells either as a result of a mutation in the *Kit* gene or following busulphan treatment (80). In addition, treatment of the host mice with GnRH did not improve the outcome (81). To date, there has been only report describing the generation of spermatozoa from transplanted human spermatogonia in approximately 25% of the host animals; however, these results are yet to be reproduced in subsequent studies and therefore should be interpreted with caution.

This failure of the host mouse testis to support spermatogenesis following SSC transplantation from larger species may be due to the failure of the host mouse somatic cells to support the development of germ cells from distant species. Therefore auto- or allo-transplantation of SSCs back into a compatible somatic environment may support normal development of the transplanted cells. Indeed, this has been demonstrated in a study involving allogenic transplantation of SSCs in adult macaque monkeys, which resulted in the generation of functional gametes capable of producing embryos. These gametes were shown to have been derived from the transplanted SSCs (82). To date, the only similar study to attempt to restore spermatogenesis in humans using this approach involved 11 men with Hodgkins lymphoma. Testis tissue was obtained prior to their cancer treatment and, in 5 patients, cell suspensions derived from these tissues were autotransplanted back into the testis following completion of their treatment (83). The outcomes in terms of restoration of spermatogenesis have not been published. Further studies involving autotransplantation of human SSCs into a compatible somatic environment are warranted to demonstrate the potential for SSC transplantation as a viable option for fertility preservation.

### Testicular tissue transplantation

Xenografting of testicular tissues into immunocompromised host mice has increasingly been used as a tool to investigate testicular development in human and non-human primates (84). This model system can be used to determine the effects of exposure to exogenous chemicals (including chemotherapy, radiotherapy and other pharmaceuticals) (71, 85) and also to investigate the role of manipulation of somatic cell signalling (e.g. exposure to hormones) on the testicular development and function in human and non-human primate testis (34, 85). Leydig cell function can be maintained in xenografts of testicular tissue from human and non-human primate as a result of stimulation by endogenous host mouse gonadotrophins, as demonstrated by a ‘basal’ level of testosterone secretion from the grafts of castrate hosts. Testosterone secretion can be significantly increased following administration of exogenous gonadotrophins (e.g. hCG) (34). Furthermore, Sertoli cell maturation is supported in xenografts of human foetal testis with or without administration of hCG to host mice (34, 86, 87). Recently, it has also been reported in prepubertal rhesus monkey testis xenografts that irradiation affects gene expression, in a dose-dependent manner, not only in germ cells but also in Sertoli and peritubular myoid cells (71).

Full spermatogenesis can be achieved in xenografts of juvenile rhesus monkey testis, and this occurs earlier (5–7 months after grafting) than it would occur *in vivo*, which may be influenced by effects on the somatic cell environment by the host mouse e.g. by stimulation of xenografts from endogenous activity of the HPG axis (88, 89). Indeed, subsequent studies have demonstrated that administration of exogenous gonadotrophins in the form of PMSG (FSH equivalent) and hCG (LH equivalent) can accelerate and sustain spermatogenesis in xenografts of infant rhesus monkey testis retrieved from castrated host mice (90). In addition, the Sertoli cells from gonadotrophin-exposed xenografts showed an increase in the proportion of tubules expressing AR, which coincided with a decrease in the proportion expressing AMH, compared to untreated control xenografts. Further evidence for maturation of the Sertoli cells was provided by investigating cell proliferation based on PCNA staining. Administration of gonadotrophins resulted in cessation of Sertoli cell proliferation indicating maturation of these cells. Taken together, these results suggest that gonadotrophins signalling through the somatic cells can induce maturation of the Sertoli cell population capable of supporting germ cell proliferation and spermatogenesis (90). Comparison of the results from these studies using testis tissue from infant and juvenile monkeys highlights potential differences in terms of Leydig cell responsiveness in testicular tissues at these two developmental stages. Exogenous hCG was able to stimulate androgen production in xenografts from both juvenile and infant monkeys. However, for host mice receiving no exogenous gonadotrophins, androgen production resulting from endogenous host mouse gonadotrophin was only demonstrated in xenografts from juvenile monkeys (88, 90).

To date, only one study has investigated the role of gonadotrophin supplementation on testicular maturation in human prepubertal testis xenografts. Exogenous FSH was administered to host mice xenografted with tissue from 6 boys aged 2.5–12 years. FSH did not promote germ cell survival and meiotic differentiation in human prepubertal testis xenografts; however, the impact of FSH on somatic cell maturation was not reported (91). In addition, these studies did not include the administration of hCG, which may be important for stimulating androgen production within the xenografts in order to support further development of the tissue. Further studies are warranted to investigate the potential for modifying the somatic cell environment to support development of human prepubertal testis xenografts.

### *In vitro* maturation of testicular tissues and cells

During the last century, tissue culture conditions for *in vitro* spermatogenesis have been developed in rodents. These studies have indicated a role for maintaining intact cell-to-cell communication pathways between somatic and germ cells to support germ cell development (92). Important aspects related to male germ cell differentiation and proliferation, for example, the positive effects of temperature below 37°C (93, 94, 95, 96, 97) or the need for functional cell–cell interactions (98, 99, 100) were reported. A smaller number of studies have been conducted using human tissues and of those only ten included the use of prepubertal tissue (summarised in Table 1).

**Table 1:** Results of studies involving *in vitro* culture of somatic cells of human prepubertal testicular tissue.

Age (years)	*n*	Clinical reason for biopsy	Culture conditions	Main findings	References
4–10	5	Cryptorchidism	Tissue culture (short-term)	*In vitro* conversion of pregnenolone (both groups) and progesterone (adult group) into testosterone	(102)
1–17	13	Prepubertal patients	Sertoli-spermatogenic co-cultures	Similar patterns of secretory proteins *in vitro*, when compared to testicular tissue. Cell viability, and differentiation potential, via synchronous DNA synthesis of preleptotene spermatocytes	(105)
1–2	7	Unilateral undescended testes	Tissue culture	No different synthesis of RNA or DNA between both groups (undescended testis and lateral control) when cultured at 31°C or 37°C	(137)
2–16	17	Unilateral undescended testes, left-sided varicocele	Tissue culture	Maximum DNA synthesis in pubertal and postpubertal testes at 31°C. Maximum DNA synthesis in prepubertal boys at 37°C. RNA and protein synthesis decreased in all three groups at 40°C and 43°C	(138)
0–3	17	Cadaveric testes	Single-cell culture	Prepubertal human testicular cells cultured for several days keeping their steroidogenic potential; cells can respond to hLH *in vitro* and their response to hrFSH might be mediated via paracrine factors. Response to human growth hormone is observed in the absence of gonadotropins	(139)
0–3	12	Cadaveric testes	Single-cell culture	Serum levels of LH, FSH, growth hormone and prolactin are higher during the first months postnatally than later in childhood	(140)
0–7	22	Cadaveric testes	Single-cell culture	*In vitro* secretion of inhibin B is related to the age of the tissue, the cells are obtained from. Newborn samples show the highest secretion potential	(141)
12–36	7	Cadaveric testes	Primary Sertoli cell cultures	Phenotypic characteristics and functionality of primary human Sertoli cells isolated from adult testes after their *in vitro* expansion could be established	(142)
15	1	Fertility preservation due to cancer treatment (pubertal boy)	Single-cell/ testicular organoid cultures on decellularised testicular scaffolds	Primary human testicular cells are able to self-organize into testicular organoids, either with or without support of testicular scaffolds. Spermatogonia and supporting somatic cells could be cultured for a period up to four weeks	(113)
2–12	3	Fertility preservation due to cancer treatment (prepubertal boy)	Tissue culture	Survival of spermatogonia *in vitro* and expression of GDNF for 139 days. Decrease of AMH and testosterone production, demonstrating maturation of Sertoli and Leydig cells, respectively	(114)

Studies performed in the 1970s using testicular tissues taken from adult men described the effect of different culture conditions on the somatic compartment of the testis. Kato and coworkers described the functional maintenance of seminiferous tubules obtained from 23 men in explant tissue culture conditions at 32°C and 5% CO_2_ for up to 56 days (93). The germ cell compartment showed a degeneration of secondary spermatocytes and early spermatids after only four days *in vitro*, whereas the number of spermatogonia did not change during the first 28 days. Interestingly, Sertoli cell numbers did not change over the whole culture period, whilst interstitial cells showed gradual transition into fibroblast-like cells.

Sertoli cell function has been demonstrated in cultures of human Sertoli cell monolayers, obtained from transsexual individuals (101). Primary cultures consisting of 95% Sertoli cells could be maintained for up to 45 days *in vitro* during which the response to FSH stimulation was maintained. Functionality of Leydig cells has also been demonstrated in cultures of testicular material from adult and prepubertal patients treated with gonadotrophins showing the conversion of pregnenolone and progesterone* in vitro* (102) as well as production of testosterone *in vitro* in testicular samples of infertile men (103). The latter study revealed the positive effect of hCG on serum testosterone levels and *in vitro* conversion of progesterone. However, in this patient, the treatment with hCG alone did not result in complete spermatogenesis unless human menopausal gonadotrohpin was added to the treatment, suggesting that testosterone alone could not initiate complete spermatogenesis. A change in Leydig cell size and number was reported to be most evident in patients showing *in vitro* conversion of progesterone. However, in cases of spermatogenic arrest with normal *in vitro* conversion of progesterone, gonadotrophin treatments did not improve spermatogenesis and/or sperm counts. The authors suggest that these cases of maturation arrest were most probably not due to impaired steroidogenesis. Therefore, the authors conclude that testicular explant cultures evaluating the *in vitro* conversion of progesterone can be used as additional diagnostic tool to evaluate the potential success of treatment protocols with gonadotrophins.

Leydig cell function has also been demonstrated in cultures of human testicular tissue, obtained from 11 normal men (age 20–31 years) and 13 men diagnosed for testicular cancer (age 20–49 years) (104). The tissue of both groups showed the conversion of pregnenolone to all steroid metabolites *in vitro*. Higher levels of estradiol, DHT, testosterone and 17-OHP were observed in cultured tissue of normal men, after stimulation with 100 ng/mL of hCG *in vitro* (104).

To assess the proliferation and differentiation potential of spermatogenic cells in children, co-cultures of human Sertoli cells with spermatogenic cells has also been performed, which showed comparable patterns of secretory proteins, when compared to intact testicular tissue (105). Testicular cells of 13 boys between 1 and 17 years of age diagnosed with cryptorchidism, were compared with cells obtained from two normal adult testes, testes of two men with prostate cancer and one man undergoing vasectomy-reversal. In addition to comparable secretory profiles, cell viability and differentiation via synchronous DNA synthesis of preleptotene spermatocytes was observed (105).

In another study, involving culture of testicular material from 12 men (age 22–38 years), FSH stimulation was able to induce human plasminogen activator (PA), a highly specific serine protease, which has an important role in the destruction and remodelling of different tissues and in cell migration. This Sertoli cell-derived factor that is believed to play a role in the blood-testis barrier and spermatogenesis in rodents, could not be observed in human testicular cell cultures employing digested cell suspensions (106). These results highlight the importance of a functioning microenvironment with intact cell–cell contacts, which may be damaged after enzymatic digestion. Therefore, preparations and the use of cell monolayers might result in less suitable culture conditions to study the regulation of spermatogenesis *in vitro*.

A number of studies have utilised non-testicular somatic cell populations to provide support to the developing germ cells. One of these studies showed the successful use of Vero cells to support human male germ cell differentiation (107). Although this study described the differentiation of round spermatids solely on morphologic criteria, a follow-up study by the same group reported, in cases of successful fertilisation, normal blastocyst formation potential (108). However, the *in vitro*-matured spermatids revealed a low fertilisation potential. In this respect, the first successful completion of meiosis and the spermiogenic process *in vitro* in humans was described in 1999 in a study that utilised seminiferous tubule cultures at 30°C (109, 110). Although morphologic analysis using the Papanicolaou method as well as fluorescence *in situ* hybridisation (FISH) and immunocytochemical detection of proacrosin 4D4 were performed to identify haploid cells generated *in vitro*, measurements of DNA contents supporting the haploid nature of the round spermatids were not performed. Despite limitations concerning the quality control of the produced sperm, further experiments on the functionality of the sperm were performed and the birth of healthy infants after ICSI using *in vitro*-differentiated sperm was reported (109). Already in 1998, the same authors highlighted the importance of the somatic environment, mainly the role of FSH and testosterone, on Sertoli and male germ cells (111). The main finding was that solely FSH induces the completion of meiosis and spermiogenesis and that testosterone has only a supporting effect on this process. The authors suggested that this was most probably due to a preventive effect of testosterone supporting Sertoli cell survival. However, testosterone alone did not initiate meiosis and spermiogenesis when added without FSH (111).

Interestingly, the differentiation of primary spermatocytes into round spermatids, which usually takes up to 32 days *in vivo* (109, 110), was reported to occur after only 48 h in this study and also in another study in which the meiotic cells were cultured on Vero cells (110, 111, 112). The explanation for this accelerated differentiation has not been elucidated but could be due to the testicular tissue used in these studies containing a small number of haploid cells prior to culture or may be due to an abnormal endocrine or paracrine interaction between the cells as a result of* in vitro* culture.

In conclusion, the search for a reproducible method, when focusing on *in vitro* differentiation of human male germ cells as well as the functionality of testicular somatic cells, clearly demonstrated that a microenvironment resembling a three-dimensional organisation of the situation *in situ* should be provided (4). The establishment of novel three-dimensional culture systems (e.g. scaffold, testicular explant or organoid culture conditions) has recently provided novel insights into the process of mammalian male germ cell differentiation and proliferation as well as formation of testicular microenvironments *in vitro* (113, 114, 115).

However, in order to establish systems for fertility preservation in humans, further studies are needed to increase our understanding of SSC niche function, formation and regulation in humans both *in vivo* and i*n vitro*. In this respect, a recent study using testicular material from 16 patients with cryptorchidism and 9 patients with obstructive azoospermia (mean ± s.e.m. 29 ± 2 years old) reported the differentiation of human SSCs up to functional haploid spermatids in a conventional single-cell culture for 7–10 days (116).

A key aspect of the studies described thus far is that it primarily involves the development of post-meiotic germ cells from human adult testis, often with spermatogenic arrest, and no similar results using prepubertal human testis tissue have been reported. In this respect, a very recent study of de Michele and coworkers reported preserved seminiferous tubule structures, along with survival of spermatogonia and Sertoli and Leydig cell maturation in cryopreserved prepubertal testis tissue cultured for up to 139 days *in vitro* (114). Testosterone production, with a peak at 10 days *in vitro* showed the functionality of Leydig cells, whilst a decrease in AMH expression after 16 days *in vitro*, suggested Sertoli cell maturation *in vitro* (114).

In addition to the concept of using explant tissue cultures, the use of cytocompatible decellularised testicular matrix (DTM) for male germ cell differentiation and testicular organoid formation has been reported in humans (113, 115, 117). These studies showed the impact of structural support, as provided by human DTM, but also demonstrated that the cells themselves can generate the necessary components without any structural support. Follow-up studies will be needed to elucidate the potential application of testicular organoids for future research.

The proteomic analysis of the DTM produced by a decellularisation protocol, employing 1% SDS treatment for 24 h, revealed that in addition to well-known components such as collagens I and IV, laminins and fibronectin, more than 100 unique proteins belonging to, or associated with testicular extracellular matrix are present (117). Therefore, the DTM was identified as a potential supportive structure, which provides a suitable matrix for testicular cells to grow and differentiate. Interestingly, a follow-up study using the DTM revealed the generation of ECM produced by the cultured cells in addition to the DTM (113). Although, testosterone and inhibin b production as well as a similar expression of cytokines, along with protein expression profiles of germ, peritubular myoid, Sertoli (including the blood-testis-barrier marker zona occludens 1) and Leydig cells could be demonstrated when using DTM as matrix, similar results could also be observed in cultures performed without addition of DTM as matrix (113). Future studies are required to investigate specific factors influencing the formation of *de novo*-formed matrices present in the testis under these culture conditions.

In parallel to the studies of Baert and coworkers, another study published recently, demonstrated the successful reorganisation of human testicular cells into testicular organoids (115). This study revealed the production of testosterone with and without hCG stimulation, also in addition to RNA expression levels for genes present in post-meiotic germ cells. In addition, experiments that generated testicular organoids demonstrated dose-response to gonadotoxic substances as busulfan, cisplatin, doxorubicin and etoposide measured by morphology in PAS stained organoids, live/dead viability assays and ATP production after 48-h incubation of testicular organoids culture for 2 or 23 days *in vitro*. Both conditions (2 and 23 days cultured testicular organoids) exhibited a dose-response decrease in viability and maintained IC_50_ values significantly higher compared to cells cultured in two-dimensional conditions (115).

Together these novel studies, highlight the potential use of testicular organoid systems for future experiments of male germ cell physiology. However, follow-up studies will be needed to elucidate the real benefit and reliability of these applications in studies on drug screening or fertility preservation methods in humans.

## Manipulation of the germ-stem cell niche during cancer treatment to preserve fertility

Whilst the majority of studies relating to protecting the testis from chemotherapy- or radiotherapy-induced damage for fertility preservation in young males focus on the direct protection of the germ cells, manipulation of the somatic cells of the SSC niche may also represent a feasible approach either as a mechanism to confer protection to the SSC or as a potential for restoration of fertility. A number of recent studies investigating the use of a variety of agents to protect the rodent testis from chemotherapy- or radiotherapy-induced testicular damage have been reported, mostly involving treatment of adult animals. This includes the use of traditional Chinese herbal compounds (118, 119), antioxidants (120, 121) and pharmaceuticals (120, 122, 123) However, to date, only a limited number of studies have investigated the association between protection of the germ cell populations and somatic cell effects.

### Sertoli cell function

Administration of L-Carnitine (LC) has been reported to result in protection of spermatogenesis in both prepubertal and adult testis when administered prior to chemotherapy (121, 124, 125). In adult mice exposed to cyclophosphamide +/− LC sperm count recovery was enhanced in those receiving LC (121). Interestingly, there was also evidence of Sertoli cell dysfunction in cyclophosphamide-exposed mice. This included reduced expression of GDNF and occludin, with increased expression of TGF-β. These alterations in Sertoli cell protein expression were prevented by the concomitant administration of LC. Whilst LC has also been shown to provide some degree of protection to germ cells in the prepubertal rodent testis, the effect of exposure on the Sertoli cells and the potential for LC to protect this somatic population has not been studied (124, 125).

Recently, a potential role for G-CSF in protecting the testis from busulphan-induced loss of spermatogenesis has been described in 5-week-old mice. Mice receiving G-CSF in addition to busulphan had significantly improved recovery of spermatogenesis compared to those receiving busulphan alone (123), and this protection was maintained over the long term (126). G-CSF has also been reported to protect the testis from irradiation-induced germ cell apoptosis in 8-week-old mice (127). CSF3R, the receptor for C-CSF, has been reported to be present on the surface of undifferentiated spermatogonia, which suggests that the protective effects may be direct via the germ cells; however, G-CSF has also an anti-inflammatory effect involving a number of cytokines, which could indicate a role for somatic cells in mediating the G-CSF-induced reduction in germ cell apoptosis (127).

### Androgen production and action

Two studies have described amelioration of androgen production or action in association with protection of the germ cell population in the adult rodent testis. Testosterone production (119) and AR (118) expression are reduced in adult mice following administration of cyclophosphamide. However, co-administration of the antioxidant *Lepidium meyenii* (Maca) or Yanjing capsule (traditional Chinese herbal preparation) were able to prevent the effects of cyclophosphamide on testosterone production and AR expression respectively.

### Manipulation of the HPG axis

A number of studies in rodents have demonstrated the potential for manipulation of the HPG axis to preserve or restore fertility in the context of chemotherapy or radiotherapy exposure (reviewed in (128)). This includes the use of GnRH agonists or antagonists in adult rats prior to treatment with procarbazine to suppress the HPG axis (129). This resulted in enhanced recovery of spermatogenesis compared to the vehicle-exposed controls. GnRH agonists administered up to 10–15 weeks after irradiation or procarbazine treatment have also resulted in an enhanced spermatogenic recovery in adult rats (129, 130). The mechanism of this protection or restoration of spermatogenesis is not clear; however, given the fact that gonadotrophins signal through the somatic cell population as described above it is likely that the effects are indirect and involve manipulation of the germ-stem cell niche. This concept is supported by results of studies in which transplantation of non-irradiated SSC into an irradiated adult rat testis did not permit resumption of spermatogenesis in the host (78); however, this could be rescued by administration of GnRH antagonists. Furthermore, subsequent studies demonstrated that introducing non-irradiated Sertoli cells into the irradiated rat testis resulted in differentiation of endogenous spermatogonia into meiotic germ cells, suggesting that donor Sertoli cells can act indirectly to support germ cell differentiation following irradiation. Whether the findings of these studies are relevant to the situation in prepuberty and to primates is important in order to determine the potential as a fertility preservation strategy in childhood cancer.

Non-human primate studies have been limited to those conducted in adult monkeys. Administration of GnRH to adult macaques in combination with irradiation (4–6.7 Gy) did not result in an increase in germ cell survival 18 months after treatment compared with irradiation alone (131, 132). Another study failed to show a protective effect of GnRHa on endogenous spermatogenesis in irradiated (7 Gy) cynomolgus monkeys (133). One study involving FSH pre-treatment of rhesus monkeys prior to receiving irradiation (1 Gy) demonstrated a significant increase in spermatogonia (A_dark_ and A_pale_) as well as a higher repopulation index (134).

Studies conducted in humans have also been restricted to adult populations and have failed to demonstrate any protective effect of manipulation of the HPG axis on chemotherapy-induced impairment of spermatogenesis (135). However, these studies involve limited numbers of patients, and in some instances, no appropriate control group were included indicating that further studies would be required to derive firm conclusions about their potential clinical use. Moreover, there have been no human studies looking at hormonal manipulation of the prepubertal testis in the context of chemotherapy or radiotherapy exposure.

Whilst it is recognised that the HPG axis is relatively quiescent during prepuberty, evidence from juvenile marmoset demonstrates that GnRH antagonist treatment can reduce testis weight, delay Sertoli cell function in terms of lumen formation and reduce Leydig cell volume, compared to controls. However, germ cell proliferation index, measured by PCNA expression in spermatogonia was not affected by GnRHa treatment, demonstrating that manipulation of the HPG axis in prepuberty can impact on somatic cell function independently of effects on germ cells despite the relatively low levels of gonadotrophins during this period of development (9). Similar reductions in testis weight, as well as a reduction in germ cell number, have also been described in neonatal/infantile marmoset following administration of GnRH antagonist from birth, compared to vehicle-exposed controls (136). This is associated with a significant decrease in germ cell proliferation in the GnRH antagonist-exposed animals ((136) and unpublished results). However, complete suppression of germ cell proliferation did not occur suggesting that spermatogonia may remain susceptible to direct damage following cancer treatment.

Taken together, the potential for manipulation of the HPG axis to protect fertility in the infantile or prepubertal human testis exposed to chemotherapy or radiotherapy remains to be determined; however, the demonstration that testicular development and function can be impacted by suppression of the HPG axis and the mechanisms by which this occurs may be of importance for developing fertility preservation strategies.

## Conclusion

Exposure to chemotherapy and radiotherapy during childhood is well known to impact on subsequent testicular function. Whilst effects of germ cells and fertility are the primary focus there are also potential impacts on the somatic cells of the testis, which may contribute to the germ cell effects as well as impacting on endocrine function. Whilst preventing direct germ cell effects remain the focus for fertility preservation strategies, manipulation of the somatic cells in the germ-stem cell niche to provide indirect protection of the germ cell population is less well described. In addition, strategies to restore fertility such as transplantation of cryopreserved SSC back into the patient after they have completed their treatment relies on the preservation of an intact somatic cell environment. Further studies are required to determine the importance of the somatic cell populations in mediating the effects of chemotherapy and radiotherapy on the testis and how this information can be used to develop strategies to preserve fertility in childhood cancer.

## Declaration of interest

The authors declare that there is no conflict of interest that could be perceived as prejudicing the impartiality of this review.

## Funding

R T M was supported by a Wellcome Trust Intermediate Clinical Fellowship (Grant no: 098522). M H was supported by EU-FP7-PEOPLE-2013-ITN 603568: 'Growsperm'.
